# Platelets as a prognostic factor for patients with adenocarcinoma of the gastroesophageal junction

**DOI:** 10.1007/s00423-023-03093-y

**Published:** 2023-09-06

**Authors:** Joy Feka, Gerd Jomrich, Daniel Winkler, Ayseguel Ilhan-Mutlu, Ivan Kristo, Matthias Paireder, Erwin Rieder, Milena Bologheanu, Reza Asari, Sebastian F. Schoppmann

**Affiliations:** 1 Department of Surgery, Medical University of Vienna, Spitalgasse 23, 1090 Vienna, Austria; 2 Department of Statistics and Operations Research, University of Vienna, Oskar Morgenstern Platz 1, 1090 Vienna, Austria; 3 Department of Medicine 1, Comprehensive Cancer Center (CCC), Medical University of Vienna, Spitalgasse 23, 1090 Vienna, Austria; 4 Upper-GI Unit, Department of Surgery, Division of General Surgery, Comprehensive Cancer Center, Medical University of Vienna, Waehringer Guertel 18-20, 1090 Vienna, Austria

**Keywords:** Adenocarcinomas of the esophagogastric junction, Neoadjuvant treatment, Primary resection, Mean platelet volume, Platelet count

## Abstract

**Objective:**

The aim of this study was to investigate the prognostic role of plasma platelet count (PLT), mean platelet volume (MPV), and the combined COP-MPV score in patients with resectable adenocarcinomas of the gastroesophageal junction.

**Background:**

Platelet activation, quantified by PLT and elevated MPV, plays an essential part in the biological process of carcinogenesis and metastasis. An increased preoperative COP-MPV is associated with poor survival in various tumor entities.

**Methods:**

Data of 265 patients undergoing surgical resection for adenocarcinoma of the gastroesophageal junction were abstracted. COP-MPV score was defined for each patient. Utilizing univariate and multivariate Cox proportional hazard analyses, survival was determined.

**Results:**

In univariate analysis, elevated PLT (HR 3.58, 95% CI 2.61–4.80, *p*<0.001) and increased COP-MPV (HR 0.27, 95% CI 0.17–0.42, *p*<0.001 and HR 0.42, 95% CI 0.29–0.60, *p*<0.001) significantly correlated with shorter patients’ overall and disease-free survival, for all 256 patients, as well as in the subgroups of neoadjuvantly treated (*p*<0.001) and primarily resected patients (*p*<0.001). COP-MPV remained a significant prognostic factor in multivariate analysis for OS. However, PLT alone showed significant diminished OS and DFS in all subgroups (*p*<0.001) in univariate and multivariate analysis.

**Conclusion:**

PLT is a potent independent prognostic biomarker for survival in a large prospective cohort of patients with resectable adenocarcinoma of the gastroesophageal junction. Additionally, we confirm that the COP-MPV score is significantly associated with worse outcome in these patients, but has no benefit in comparison to PLT.

## Introduction

Esophageal cancer ranks as the sixth leading cause of cancer-related deaths worldwide [1–4]. Whereas the occurrence of squamous cell carcinoma (SCC) is declining, the rise in incidence of adenocarcinoma (AC) of the esophagogastric junction (AEG) in Western countries has become alarming [5, 6]. This increase in incidence is mainly explained by the risk factors for esophageal adenocarcinoma: gastroesophageal reflux disease (GERD) resulting in Barrett esophagus and increased obesity and waist circumference, which are highly represented in Western countries [7].

Treatment options vary according to tumor stage: patients might undergo neoadjuvant chemotherapy prior to surgery or receive primary resection and then followed by postoperative therapy [6, 8–10]. However, despite new developments of treatment protocols and new therapeutic approaches, the prognosis of these patients is still poor, with a 5-year survival rate of 20% [4, 10]. Factors that predict prognosis are only available after surgery, such as tumor and lymph node staging, tumor histology, and resection margin status [4]. Several other biomarkers have already been found, which can be detected prior surgery, but lacked in power or feasibility to be implemented into the clinical setting [10]. Hence, this illustrates the necessity of detecting effective and easy-obtained biomarkers for prognosis.

In the last decade, several studies have suggested that platelet activation plays an essential role in the biological process of carcinogenesis and metastasis [11–13]. Platelet activation is quantified by two parameters that are generally measured for routine analysis during a complete blood count: platelet count (PLT) and mean platelet volume (MPV) [14]. Irregular platelet production and activation can be identified due to elevated MPV levels, which have been proven increased in malignant tumors, such as colorectal, lung, ovarian, or gastric cancer [15–18]. Recent studies have evaluated the prognostic value of the COP-MPV score, a new tool that incorporates both PLT and MPV.

The COP-MPV score is calculated as follows: Patients with both elevated PLT and MPV values receive a score of 2. If one value is elevated, one COP-MPV point is given. If none are elevated, the score is 0. The cut-off values were determined by the mean value via dividing the sum of the values by the number of values. The mean values of PLT and MPV are 286 G/l and 10.1 fl.

An elevated COP-MPV score has already proven to be significantly associated with poor survival for non-small cell lung cancer and esophageal squamous cell cancer [19–22].

However, studies have not yet investigated the prognostic role of COP-MPV in patients suffering from esophagogastric adenocarcinoma.

The aim of this study was to investigate the prognostic role of the COP-MPV score in 265 patients, who underwent neoadjuvant treatment prior to surgery or solely primary resection of the adenocarcinoma of the esophagogastric junction.

## Material and methods

### Patient collective

Consecutive patients from a prospective database that received curative esophageal resection for adenocarcinoma (AEG) in the period of January 1992 and April 2016 were included in this retrospective analysis. The database has been built throughout the years by collecting essential information about every patient receiving an esophageal resection in the department of general surgery dated back since 1992. This study (EK1652/2016) was approved by the Institutional Review Board of the Medical University of Vienna, Austria, according to the declaration of Helsinki. Exclusion criteria were defined as patients with malignancies other than AEG, distant metastasis, missing preoperative laboratory values, positive resection margin, and death within 30 days postoperatively. Patients’ data, such as demographical, clinicopathological, histopathological, and laboratory values, were collected in a prospective database.

Patients with locally advanced AEG received neoadjuvant chemotherapy, either oxaliplatin/capecitabine or cisplatin-/5-fluorouracil-based regiments, standardized by the Comprehensive Cancer Center of the Medical University of Vienna. Tumor regression grade due to neoadjuvant chemotherapy was classified as defined by Mandard et al. [23]. The clinical tumor stage was determined according to the pathological tumor-node-metastasis (TNM) classification of the Union for International Cancer Control (UICC), 8th edition [24, 25]. The classification of the adenocarcinoma was dependent on location in accordance to the Siewert and Stein classification [6].

### Surgery

Esophagogastric junction (EGJ) cancer can be subcategorized according to Siewert and Stein: If the epicenter of the carcinoma is located 1–5 cm above the EGJ, it is classified as AEG I. AEG II is characterized as located epicenter of 1 cm above and 2 cm below the EGJ. If the cancer can be found 2–5 cm below the EGJ, it is labelled AEG III [6].

According to the current guidelines, patients diagnosed with AEG I underwent en bloc abdominothoracic esophagectomy, whereas patients located at the AEG III position received transhiatal extended gastrectomy. In patients with AEG II tumors, the extent of resection was decided individually.

Postoperatively, patients received follow-up controls at the outpatient clinic every 3 months for the first 2 years and then every 6 months for 3 more years including CT scan and blood examination with tumor markers. After 5 years without reoccurrence, no further examination was necessary.

### Blood examinations

Laboratory values were acquired within a period of 3 days prior to the commencement of neoadjuvant therapy or prior to surgery in patients who underwent primary resection. Platelet count (PLT) and mean platelet volume (MPV) were evaluated in all patients. All included patients did not show any signs of fever (>37.2 °C), infection, or chronic inflammatory disease at the time point of blood draw. The Department of Laboratory Medicine, Medical University of Vienna, which is the central laboratory of the General Hospital of Vienna, determined these parameters.

### Statistical analysis

Overall survival (OS) was defined as the time between surgery and the death (from any cause) of the patient. Disease-free survival (DFS) on the other hand determines the time between surgery and the progression of the malignancy. The start of the observation period for neoadjuvantly treated patients was defined as the date of the first blood draw 1 day prior start of chemotherapy. The end of the observation period was considered the date of last alive contact, if there was no indication that the patient had died before that time point.

Categorical variables were shown as frequency (percentage), while continuous variables were presented as the mean values ± standard deviation. Unpaired *t* or *χ*^2^ test was used to compare whether statistical differences between groups were significant. Univariate analyses were used to narrow down the list of possible prognostic factors. Significant factors were then brought into multivariate Cox proportional hazard model to determine their independency. The Kaplan-Meier curves and log-rank test were used to compare survival differences among groups. All statistical analyses were performed using the statistical software R version 3.44 (Vienna, Austria). Statistical significance was defined as a *p*-value < 0.05.

### COP-MPV scoring

The optimal cut-off values of PLT and MPV were 286 G/l and 10.1 fl, respectively. The COP-MPV score was then calculated on the basis of the median value of these two platelet characteristics. Patients were divided into three groups: a COP-MPV score of 2 was given to patients with both a higher platelet count (≥286 G/l) level and a higher mean platelet volume (≥10.1 fl). Patients who had one of these two values elevated received a COP-MPV score of 1, whereas patients with neither higher value were grouped a score of 0.

## Results

At the Department for Surgery of the Medical University of Vienna, 544 patients were diagnosed with resectable AEG between 1992 and 2016, and due to missing data, a total of two hundred and sixty-five patients were included in this study.

In this collective, 216 patients (81.5%) were male, 49 (18.5%) were female, and 63 patients were lost to follow-up. The mean age was 63.6 years, with a standard deviation of 10.9 years. The ethnicity distribution of all study patients could not be analyzed due to the retrospective character of this study. One hundred two patients (38.5%) were neoadjuvantly treated; the other 163 patients (61.5%) received primary resection. When classified into the AEG definition, 163 patients (61.5%) were diagnosed with AEG I, 79 patients (29.8%) were with AEG II, and 23 patients (8.7%) were in the AEG III group. (For a detailed characterization of the study cohort, please see Table 1.)

**Table 1:** Clinical characteristics between different COP-MPV groups

	COP-MPV score					
	0		1		2	
Age (± SD)	62.6 (± 10.5)		64.2 (±10.5)		64.0 (±11.5)	
	*n*	*%*	*n*	*%*	*n*	*%*
Collective	65	24.5	139	52.5	61	23
Female	12	4.5	25	9.4	12	4.5
Male	53	20	114	43	49	18.5
G						
0	3	1.1	-	-	1	0.3
1	2	0.8	3	1.1	1	0.3
2	26	9.8	67	25.3	18	6.8
3	34	12.8	69	26	41	15.5
AEG						
I	42	15.8	87	32.8	34	12.8
II	17	6.4	37	14	25	9.4
III	6	2.3	15	5.7	2	0.8
Neoadjuvant chemotherapy						
Yes	25	9.4	42	15.8	35	13.2
No	40	15.5	97	36.6	26	9.8
Overall survival						
3-year OS	45.3%					
5-year OS	26.8%					

*AEG* adenocarcinoma of the esophagogastric junction, *COP-MPV* combination of plasma platelet count and mean platelet volume, *G* histological grade, *OS* overall survival

Applying the median values of PLT and MPV (286 G/l and 10.1 fl, respectively) as a cutoff, the cohort was divided into COP-MPV subgroups. The grouping was as follows: 65 patients (24.5%) were in the COP-MPV score 0 group, 139 patients (52.5%) were scored COP-MPV-1, and 61 patients (23%) were in the score 2 group.

**Fig. 1:**
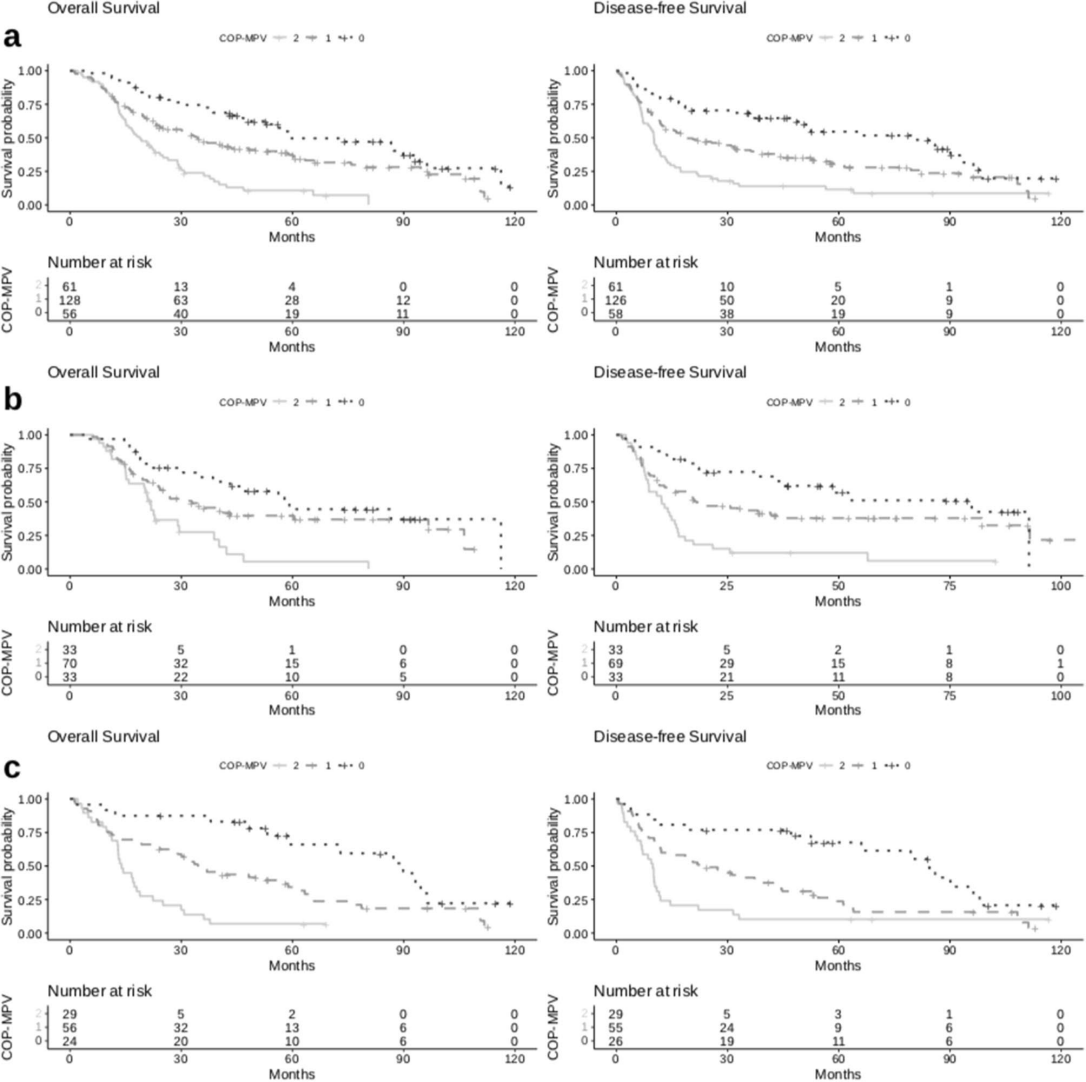
Kaplan-Meier curves of overall survival and disease-free survival for **a** all patients, **b** 102 patients who received neoadjuvant treatment, and **c** 163 patients who underwent primary resection. COP-MPV, combination of plasma platelet count and mean platelet volume

OS of the complete cohort was of 45.3% at 3 and 26.8% at 5-year follow-up, respectively. The median OS for patients with COP-MPV score 0 group was 56.1 months, 32.8 months in the score 1 group, and 18.8 months in the score 2 group. When reviewing the COP-MPV group individually, the highest survival rate was seen in the COP-MPV group with a score of 0 with 72.3% in 3 years and 43.1% in 5 years. Patients with a COP-MPV score of 1 had a 3-year survival of 44.6% and a 5-year survival of 28.1%. The lowest survival rates were seen in the COP-MPV group with a score of 2. These patients had a 3- and 5-year overall survival of 18% and 6.6%. The Kaplan-Meier survival analysis showed that high PLT and high MPV were associated with poor OS and DFS for all the patients (all *p* < 0.001) (Fig. 1a).

In univariate analyses, platelet count (*p*<0.001), COP-MPV score 0 (*p*<0.001), COP-MPV score 1 (*p*<0.001), clinical tumor stage (*p* < 0.001), preoperative UICC classification (*p* < 0.001), grading (*p* < 0.001), pT (*p* < 0.001), pN (*p* < 0.017), lymph-node ratio (*p* < 0.001), Mandard response 3 (*p*=0.033), and neoadjuvant therapy (yes/no) (*p*<0.001) were significantly associated with OS. Similar results were shown in association with DFS regarding these parameters (Table 2). Some parameters showed no significant correlation to OS, such as MPV level, sex, age, nor ASA.

**Table 2:** Univariate analyses of overall survival and disease-free survival for all patients

	Overall survival				Disease-free survival			
	HR	95% CI		π	HR	95% CI		π
Sex	0.92	0.62	1.37	0.669	0.98	0.66	1.45	0.918
Age	1.00	0.99	1.02	0.679	1.00	0.99	1.01	0.941
PLT	3.54	2.58	4.84	<0.001	3.07	2.26	4.18	<0.001
MPV	1.02	0.76	1.37	0.908	1.09	0.81	1.47	0.554
COP-MPV (vs. 2)								
0	0.26	0.28	0.40	<0.001	0.28	0.18	0.43	<0.001
1	0.39	0.28	0.57	<0.001	0.45	0.32	0.63	<0.001
UICC preOP (vs. III)								
I	0.50	0.29	0.87	0.013	0.41	0.23	0.73	0.002
IIA	0.75	0.44	1.30	0.308	0.77	0.45	1.31	0.333
IIB	0.54	0.33	0.88	0.015	0.57	0.35	0.93	0.026
IVA	1.78	1.15	2.76	0.009	1.73	1.13	2.66	0.012
UICC postOP (vs. IIIB)								
I	0.46	0.25	0.83	0.011	0.41	0.23	0.73	0.002
IB	0.14	0.07	0.30	<0.001	0.77	0.45	1.31	0.333
IC	0.42	0.23	0.76	0.004	0.57	0.35	0.93	0.026
II	0.77	0.44	1.35	0.362	1.73	1.13	2.66	0.012
IIA	0.48	0.14	1.56	0.220	0.77	0.45	1.31	0.333
IIB	0.47	0.20	1.09	0.009	1.73	1.13	2.66	0.012
IIIA	0.80	0.50	1.28	0.308	0.77	0.45	1.31	0.333
IVA	1.55	0.98	2.45	0.063	1.73	1.13	2.66	0.012
cT (vs. 3)								
1	0.56	0.40	0.78	<0.001	0.42	0.28	0.65	<0.001
2	0.47	0.31	0.71	<0.001	0.56	0.40	0.77	<0.001
cN (vs. I)								
0	0.60	0.43	0.84	<0.001	0.55	0.39	0.77	<0.001
2	1.92	1.21	3.03	<0.001	1.67	1.06	2.64	0.027
3	1.22	0.39	3.85	0.734	1.94	0.71	5.29	0.194
pT (vs. 3)								
0	0.85	0.31	2.33	0.757	0.34	0.08	1.36	0.126
1	0.26	0.16	0.42	<0.001	0.28	0.18	0.44	<0.001
2	0.72	0.50	1.02	0.065	0.66	0.47	0.95	0.023
4	1.44	0.75	2.77	0.278	1.30	0.68	2.50	0.431
pN (vs. O)								
1	2.41	1.69	3.42	<0.001	2.51	1.76	3.58	<0.001
2	2.76	1.69	4.51	<0.001	2.98	1.84	4.82	<0.001
3	4.42	2.63	7.45	<0.001	4.82	2.92	7.95	<0.001
AEG (vs. 1)								
II	1.14	0.83	1.57	0.420	1.10	0.80	1.51	0.564
III	0.65	0.35	1.18	0.152	0.62	0.33	1.16	0.133
G (vs. 3)								
0	0.91	0.29	2.86	0.869	0.62	0.15	2.51	0.503
1	0.48	0.17	1.31	0.150	0.52	0.19	1.40	0.195
2	0.46	0.33	0.63	<0.001	0.50	0.36	0.69	<0.001
Mandard response* (vs. 5)								
1	1.78	0.28	2.22	0.647	0.31	0.07	1.28	0.106
2	0.41	0.16	1.06	0.065	0.37	0.15	0.94	0.036
3	0.51	0.27	0.95	0.033	0.34	0.18	0.66	0.001
4	0.65	0.39	1.09	0.089	0.64	0.40	1.03	0.063
Neoadjuvant chemotherapy								
Yes (vs. no)	2.31	1.71	3.11	<0.001	2.16	1.60	2.91	<0.001
ASA (vs. 2)								
1	1.06	0.72	1.53	0.775	1.20	0.83	1.74	0.325
3	1.29	0.81	2.05	0.287	1.29	0.82	2.04	0.271
4	0.92	0.22	3.72	0.905	0.44	0.06	3.14	0.411

*p*-value < 0.05; *AEG* adenocarcinoma of the esophagogastric junction, *ASA* American Society of Anesthesiologists, *CI* confidence interval, *COP-MPV* combination of plasma platelet count and mean platelet volume, *HR* hazard ratio, *G* histological grade, *MPV* mean platelet volume, *PLT* plasma platelet count, *UICC* Union for International Cancer Control

*Mandard response only applicable for neoadjuvant treated patients

Multivariate analyses for the whole cohort demonstrated that COP-MPV score 0 (*p*=0.001), COP-MPV score 1 (*p*=0.001), and PLT (*p*<0.001) were independent prognostic factors for OS (Table 3).

**Table 3:** Multivariate analyses of overall survival in all patients, neoadjuvantly treated patients, and primarily resected patients

Overall survival	All patients				Neoadjuvantly treated				Primarily resected			
	*HR*	*95% CI*		*π*	*HR*	*95% CI*		*π*	*HR*	*95% CI*		*π*
PLT	3.72	2.67	5.18	<0.001	0.21	0.12	0.37	<0.001	3.18	1.96	5.17	<0.001
Sex	0.96	0.64	1.46	0.856	0.52	0.25	1.09	0.840	1.66	0.96	2.88	0.070
Age	1.00	0.99	1.02	0.645	1.01	0.99	1.03	0.410	1.01	0.99	1.03	0.229
G (vs. 3)												
0	1.53	0.45	5.22	0.501	0.57	0.05	6.19	0.650	-	-	-	-
1	0.43	0.15	1.21	0.109	3.44	0.37	32.23	0.281	0.48	0.14	1.71	0.258
2	0.62	0.44	0.89	0.009	0.85	0.50	1.45	0.553	0.53	0.32	0.88	0.013
UICC preOP (vs. III)												
I	0.90	0.49	1.67	0.746	0.95	0.29	3.09	0.934	1.31	0.48	3.56	0.592
IIA	1.06	0.60	1.88	0.841	1.78	0.71	4.47	0.217	1.15	0.48	2.73	0.750
IIB	0.75	0.45	1.28	0.293	1.46	0.64	3.30	0.367	0.91	0.36	2.24	0.824
IVA	1.21	0.77	1.91	0.405	1.27	0.66	2.46	0.472	1.40	0.73	2.70	0.312
Mandard response (vs. 5)												
1	-	-	-	-	2.44	0.23	25.65	0.458	-	-	-	-
2	-	-	-	-	0.58	0.20	1.69	0.320	-	-	-	-
3	-	-	-	-	1.04	0.52	2.07	0.912	-	-	-	-
4	-	-	-	-	1.11	0.63	1.95	0.715	-	-	-	-
MPV	1.05	0.78	1.42	0.752	1.17	0.73	1.87	0.520	1.59	1.02	2.47	0.039
Sex	1.11	0.73	1.69	0.614	0.68	0.32	1.47	0.326	1.81	1.07	3.08	0.028
Age	1.00	0.99	1.02	0.486	0.99	0.97	1.02	0.612	1.03	1.00	1.05	0.019
G (vs. 3)												
0	1.18	0.34	4.02	0.794	0.70	0.06	8.04	0.775	-	-	-	-
1	0.53	0.19	1.46	0.217	9.36	1.02	86.02	0.048	0.74	0.22	2.56	0.64
2	0.55	0.39	0.77	0.001	0.58	0.34	0.97	0.038	0.65	0.41	1.03	0.07
UICC preOP (vs. III)												
I	0.74	0.41	1.33	0.313	1.07	0.35	3.29	0.912	1.53	0.56	4.17	0.403
IIA	0.95	0.54	1.69	0.864	1.51	0.59	3.88	0.389	1.78	0.76	4.21	0.186
IIB	0.68	0.40	1.13	0.136	0.93	0.40	2.13	0.860	1.15	0.45	2.94	0.773
IVA	1.40	0.89	2.18	0.144	1.35	0.71	2.54	0.358	1.77	0.91	3.44	0.093
Mandard response (vs. 5)												
1	-	-	-	-	1.06	0.10	10.73	0.963	-	-	-	-
2	-	-	-	-	0.59	0.20	1.79	0.353	-	-	-	-
3	-	-	-	-	0.67	0.33	1.34	0.252	-	-	-	-
4	-	-	-	-	0.76	0.44	1.32	0.338	-	-	-	-
COP-MPV (vs. 2)												
0	0.29	0.19	0.45	<0.001	0.32	0.16	0.64	0.001	0.21	0.11	0.42	<0.001
1	0.54	0.37	0.79	0.001	0.41	0.24	0.71	0.001	0.52	0.30	0.90	0.019
Sex	1.11	0.74	1.67	0.621	0.71	0.33	1.51	0.372	1.71	0.99	2.95	0.051
Age	1.00	0.99	1.02	0.478	1.00	0.98	1.02	0.841	1.02	1.00	1.04	0.036
G (vs. 3)												
0	1.49	0.44	5.13	0.523	0.61	0.05	7.16	0.692	-	-	-	-
1	0.56	0.20	1.57	0.268	8.27	0.86	79.18	0.067	0.66	0.19	2.34	0.519
2	0.56	0.39	0.79	0.001	0.59	0.35	0.98	0.041	0.60	0.37	0.97	0.037
UICC preOP (vs. III)												
I	0.80	0.44	1.45	0.455	1.11	0.36	3.44	0.853	1.55	0.57	4.23	0.393
IIA	0.99	0.56	1.77	0.992	1.86	0.72	4.82	0.204	1.59	0.68	3.74	0.285
IIB	0.76	0.45	1.28	0.300	1.31	0.57	3.03	0.531	1.00	0.40	2.53	0.994
IVA	1.36	0.87	2.14	0.183	1.14	0.59	2.18	0.702	1.74	0.90	3.38	0.101
Mandard response (vs. 5)												
1	-	-	-	-	1.15	0.11	11.58	0.905	-	-	-	-
2	-	-	-	-	0.53	0.18	1.52	0.236	-	-	-	-
3	-	-	-	-	0.63	0.32	1.23	0.173	-	-	-	-
4	-	-	-	-	0.82	0.47	1.43	0.488	-	-	-	-

*p*-value < 0.05; *CI*, confidence interval; *COP-MPV*, combination of plasma platelet count and mean platelet volume; *HR*, hazard ratio; *MPV*, mean platelet volume; *PLT*, plasma platelet count

In the subgroup analysis of neoadjuvantly treated patients, elevated PLT and a COP-MPV score of 0 and 1 (vs. 2), UICC stages, and G2 were associated with a diminished OS and DFS as well. The Cox regression analysis identified PLT (*p*<0.001), COP-MPV score 0 (*p*<0.001), and COP-MPV score 1 (*p*<0.001) as significant prognostic factors for OS and DFS for neoadjuvantly treated patients (Tables 3 and 4). These findings have been visualized by the Kaplan-Meier curves (Fig. 1b).

**Table 4:** Univariate analyses of overall survival and disease-free survival for patients who received neoadjuvant treatment

	Overall survival				Disease-free survival			
	HR	95% CI		π	HR	95% CI		π
Sex	0.59	0.29	1.18	0.132	0.57	0.28	1.13	0.109
Age	0.99	0.98	1.02	0.731	0.99	0.98	1.01	0.615
PLT	0.25	0.15	0.39	<0.001	0.26	0.17	0.41	<0.001
MPV	1.24	0.81	1.89	0.313	1.02	0.67	1.55	0.921
COP-MPV (vs. 2)								
0	0.32	0.17	0.59	<0.001	0.27	0.15	0.50	<0.001
1	0.44	0.26	0.72	<0.001	0.42	0.26	0.68	<0.001
UICC preOP (vs. III)								
I	0.87	0.37	2.02	0.741	0.65	0.26	1.62	0.354
IIA	1.59	0.68	3.71	0.283	1.55	0.66	3.60	0.311
IIB	0.91	0.44	1.87	0.794	0.97	0.48	1.97	0.936
IVA	1.56	0.86	2.83	0.141	1.58	0.89	2.80	0.120
UICC postOP (vs. IIIB)								
I	0.61	0.32	1.17	0.137	0.65	0.29	1.09	0.089
II	1.04	0.56	1.91	0.910	0.88	0.47	1.64	0.690
IIIA	0.91	0.43	1.91	0.800	0.92	0.44	1.91	0.813
IVA	1.87	1.01	3.47	0.047	1.99	1.12	3.56	0.019
cT (vs. 3)								
1	0.94	0.50	1.77	0.850	0.80	0.42	1.54	0.509
2	0.73	0.45	1.19	0.204	0.77	0.48	1.23	0.276
cN (vs. 1)								
0	0.83	0.51	1.33	0.437	0.70	0.43	1.14	0.149
2	1.51	0.81	2.81	0.192	1.27	0.69	2.35	0.443
3	0.90	0.12	6.55	0.914	3.91	0.93	16.49	0.064
pT (vs. 3)								
0	1.02	0.37	2.81	0.975	0.44	0.11	1.80	0.250
1	0.63	0.28	1.38	0.245	0.67	0.32	1.40	0.285
2	0.73	0.41	1.29	0.281	0.65	0.37	1.16	0.146
4	1.31	0.53	3.29	0.559	1.11	0.45	2.78	0.817
pN (vs. 0)								
1	1.32	0.81	2.16	0.271	1.43	0.86	2.37	0.169
2	1.11	0.54	2.28	0.783	1.44	0.73	2.85	0.298
3	2.76	1.42	5.40	0.003	3.31	1.76	6.23	0.001
AEG (vs. 1)								
II	1.17	0.67	2.03	0.574	0.97	0.55	1.70	0.915
III	0.77	0.38	1.59	0.484	0.69	0.33	1.45	0.329
G (vs. 3)								
0	0.95	0.29	3.01	0.918	0.69	0.17	2.83	0.607
1	7.51	0.98	57.33	0.052	2.58	0.35	18.89	0.350
2	0.50	0.31	0.81	0.005	0.64	0.41	1.00	0.051
Mandard response (vs. 5)								
1	0.78	0.28	2.21	0.647	0.31	0.07	1.28	0.106
2	0.41	0.16	1.06	0.065	0.37	0.15	0.94	0.036
3	0.51	0.27	0.95	0.033	0.34	0.18	0.66	0.001
4	0.65	0.39	1.07	0.089	0.64	0.39	1.03	0.063
ASA (vs. 2)								
1	1.31	0.82	2.10	0.265	1.57	0.99	2.50	0.058
3	1.06	0.52	2.16	0.881	1.12	0.57	2.20	0.751
4	2.43	0.59	10.05	0.221	1.01	0.14	7.30	0.995

*p*-value < 0.05; *AEG*, adenocarcinoma of the esophagogastric junction; *ASA*, American Society of Anesthesiologists; *CI*, confidence interval; *COP-MPV*, combination of plasma platelet count and mean platelet volume; *HR*, hazard ratio; *G*, histological grade; *MPV*, mean platelet volume; *PLT*, plasma platelet count; *UICC*, Union for International Cancer Control

In the subgroup analysis of patients undergoing primary resection of the adenocarcinoma, PLT (*p*<0.001), MPV (*p*=0.036), COP-MPV score 0 (*p*<0.001), COP-MPV score 1 (*p*<0.001), postoperative UICC classification (*p* < 0.001), grading (*p* < 0.001), and surgery technique (one/two cavity procedures) (*p*=0.022) showed in univariate analysis a reduced OS and DFS (Table 5). These findings remained independent prognostic factors for OS in multivariate analysis: PLT (*p*<0.001), MPV (*p*=0.039), COP-MPV score 0 (*p*<0.001), and COP-MPV score 1 (*p*=0.019) (Table 3). The Kaplan-Meier curves emphasize these findings (Fig. 1c).

**Table 5:** Univariate analyses of overall survival and disease-free survival for patients who underwent primary resection

	Overall survival				Disease-free survival			
	HR	95% CI		π	HR	95% CI		π
Sex	1.22	0.74	2.01	0.438	1.44	0.88	2.36	0.143
Age	1.01	0.99	1.04	0.227	1.01	0.99	1.03	0.374
PLT	3.07	1.99	4.72	<0.001	2.42	1.58	3.69	<0.001
MPV	1.57	1.03	2.39	0.036	1.65	1.08	2.52	0.0197
COP-MPV (vs. 2)								
0	0.15	0.08	0.29	<0.001	0.20	0.11	0.39	<0.001
1	0.30	0.18	0.29	<0.001	0.38	0.23	0.63	<0.001
UICC preOP (vs. III)								
I	0.36	0.18	0.73	0.005	0.30	0.14	0.64	0.001
IIA	0.53	0.26	1.07	0.077	0.54	0.27	1.07	0.075
IIB	0.35	0.17	0.70	0.003	0.38	0.19	0.76	0.006
IVA	0.87	1.19	4.33	0.012	2.04	1.07	3.88	0.030
AEG (vs. 1)								
II	1.25	0.82	1.91	0.306	1.28	0.84	1.96	0.247
III	0.45	0.14	1.45	0.179	0.49	0.15	1.57	0.230
G (vs. 3)								
1	0.35	0.11	1.15	0.084	0.37	0.11	0.18	0.093
2	0.42	0.27	0.65	<0.001	0.41	0.26	0.63	<0.001
ASA (vs. 2)								
1	0.65	0.32	1.31	0.232	0.71	0.35	1.42	0.332
3	1.60	0.86	2.96	0.134	1.52	0.82	2.81	0.183

*p*-value < 0.05; *AEG*, adenocarcinoma of the esophagogastric junction; *ASA*, American Society of Anesthesiologists; *CI*, confidence interval; *COP-MPV*, combination of plasma platelet count and mean platelet volume; *G*, histological grade; *HR*, hazard ratio; *MPV*, mean platelet volume; *PLT*, plasma platelet count; *UICC*, Union for International Cancer Control

## Discussion

The significance of platelet activation in cardiovascular diseases has already been proven [26, 27].

Nowadays, the focus lies on determining the role of platelets in malignant diseases. Various studies have shown significantly elevated platelet activation in up to 10 to 57% of cancer patients [28–31]. The platelet activation can be caused by numerous factors, for example, cytokine mediation or secretion of soluble mediators like ADP, thromboxane A2 (TXA2), or high-mobility group box 1 (HMGB1) by tumor cells [31–33]. Platelets play a crucial role in the regulation of the innate and adaptive immune system, in the activation status of the endothelium, and in tumor cell proliferation and extravasation [34–37]. This elevation can cause higher cancer-associated mortality by increased tumor growth and accelerate metastasis as well as elevated risk for thrombosis and hypercoagulation [38]. This emphasizes the further needed exploration of the tumor microenvironment [25]. Platelets (PLT) are also known to play a crucial role in the regulation of tumorigenesis and tumor progression, including in cases of gastric cancer and colorectal cancer, among others [39–41]. It has been shown that PLT has the ability to induce the epithelial-mesenchymal transformation (EMT) of malignant tumors through the secretion of TGF-β and promote angiogenesis, tumor progression, and metastasis [42, 43]. Furthermore, it has been discovered that PLT-generated PD-L1 can promote tumor cells lacking PD-L1 expression to evade immune surveillance and T-cell elimination, leading to the progression of malignant tumors [43]. All of this evidence suggests that platelets have both prognostic and immunotherapeutic values. A current focus of research is the investigation of platelet-related signaling pathways in various cancers and their possible effect on immune checkpoint inhibitor therapy [44, 45]. At present, no differentiation between these COP-MPV subgroups is made in the clinical setting, but patients with a higher level of platelet activation may benefit from a personalized, targeted therapy in the future.

Platelet activation can be visualized by PLT and MPV [14]. High PLT levels are associated with lower overall survival in various cancer entities [46, 47]. The relationship of prognostic validity and MPV, which is considered to be the hallmark of platelet activation, is still discussed [48, 49]. Some studies, however, report worse survival rates in patients with higher MPV plasma levels [50]. Therefore, a new scoring tool was introduced in order to combine these two parameters and assess a possible association of elevated platelet activation and diminished survival rates: the COP-MPV score. This score classifies patients into three categories: patients with a high PLT and MPV level receive a score of 2; if one of the parameters is elevated, the patients receive 1; and if both values are in normal rage, the patients receive a score of 0 [19]. Park et al. and Zhang et al. were able to detect that high COP-MPV scores are a prognostic factor in patients suffering from oral and esophageal squamous cell carcinoma [19, 22]. Similar results have also been published by Gao et al. for non-small cell lung cancer [21]. In cases of head and neck cancer, Tham et al. were not able to detect any correlation between high COP-MPV scores and diminished survival rates [20]. Building upon these findings, this study was conducted to evaluate the COP-MPV score on patients suffering from adenocarcinoma of the esophagogastric junction, who underwent surgery in our clinic.

Here, we show that both COP-MPV and PLT alone are statistically significant in predicting prognosis in the 265 patients enrolled in this study. A high COP-MPV score is associated with diminished overall and disease-free survival of all the patients (*p*<0.001). This result is also representable in patients who received neoadjuvant treatment (*p*<0.001) as well as in patients who underwent primary resection (*p*<0.001). When investigating the significance of these values individually, MPV is not associated with the prediction of prognosis in the whole patient cohort. However, high PLT values are significantly correlated with diminished overall and disease free survival in the whole patient cohort and its subgroups (*p*<0.001).

These findings suggest that although COP-MPV is significantly associated with worse outcome in patients with gastroesophageal junction adenocarcinoma, there is no need to combine MPV and PLT into this new prognostic tool, as PLT alone is as effective as COP-MPV in predicting the prognosis of AEG patients. Although this study demonstrates that COP-MPV and PLT alone are a prognostic factor for patients with adenocarcinoma of the esophagogastric junction, there are some limiting factors that should be mentioned. Due to the fact that this study was conducted retrospectively, there may be a selection bias due to partial accessibility of laboratory results. Also, some patients had to be excluded, because the differential blood count was missing for patients who underwent resection during our defined time period. Another limiting factor is that this was conducted as a single-center experience.

## Conclusion

In this analysis, we were able to define PLT as an independent prognostic biomarker for overall survival and disease-free survival in patients with resectable adenocarcinomas of the gastroesophageal junction with or without neoadjuvant treatment. Further studies have to be conducted in order to find novel ways for early tumor detection, new treatment options, and characterization of potent biomarkers. Thus, improving the prognosis of this cancer.
